# Prevalence and predictors of admission at the time of presentation in first episode psychosis

**DOI:** 10.1007/s00127-023-02552-7

**Published:** 2023-09-03

**Authors:** Louisa Gannon, Eddie Mullen, Patrick McGorry, Brian O’Donoghue

**Affiliations:** 1 Department of Psychiatry, University College Dublin, Ireland, St Vincent’s University Hospital, Elm Park, Dublin 4, Ireland; 2 Orygen, Melbourne, VIC Australia; 3 Centre for Youth Mental Health, University of Melbourne, Melbourne, Australia

**Keywords:** First episode psychosis, Admission rates, Involuntary, Psychotic disorders

## Abstract

**Background:**

Individuals presenting with first episode psychosis (FEP) constitute a population with high admission rates. Across psychiatric services, community based treatment is aimed for where appropriate. Therefore, further knowledge on predictors of admission is required.

**Purpose:**

The objectives were to: (i) determine the proportion of individuals with FEP admitted at time of presentation (voluntarily and involuntarily) (ii) identify associated demographic and clinical factors.

**Methods:**

This study included all young people (aged 15–24) who presented with FEP to the Early Psychosis Prevention and Intervention Centre, Melbourne, Australia from 01.01.11 to 31.12.16. Binary logistic regression was used to determine unadjusted and adjusted odds ratios.

**Results:**

Of 1208 participants, 58.6% were male and the median age was 20 years (I.Q.R.17–22). At time of presentation, 50.2% were admitted. On multivariate analysis, the following factors predicted admission: being a migrant (OR = 1.75, 95% CI [1.17, 2.62]), aggression (OR = 1.42, 95% CI [1.02, 1.99]), and more severe psychotic symptoms. Longer duration of untreated psychosis was associated with lower admission rates. 70.1% of admissions were involuntary (33.7% of the cohort). Risk factors for involuntary admission were consistent with any admission, other than aggression, and with the addition of older age and male sex.

**Conclusion:**

There remains a high admission rate for FEP, even in an established early intervention service, with severity of psychopathology being the strongest predictive factor. There is an independent association between migrancy and admission. Potential reasons for these findings are discussed, and initiatives to reduce admission rates including (i) interventions to prevent admission and (ii) alternative care pathways.

## Introduction

There is an overarching aim to support individuals affected by mental health disorders in their homes if appropriate. Those presenting with a first episode of psychosis (FEP) constitute a clinical population in which there are high rates of hospital admission [1]. Within two years of diagnosis, over one third (35%) of individuals with a FEP are admitted to hospital [2] and this increases to over half (55%) within seven years [3].

There are benefits to admission and it may be necessary in certain cases. For patients presenting with high risk, it can offer a protective environment. It can help with medication adherence and can also provide a relative distance to problematic circumstances for many. However, admission is also associated with costs, both personal and economic. For the individual, admission can be more restrictive, and this has relevance for the management of psychotic disorders, as the peak age of onset is during late adolescence or early adulthood, a key developmental period. Young people who were admitted to hospital report that it can disrupt their education and vocational goals and that it can be a traumatic experience [4, 5]. From an economic perspective, approximately half of the financial costs of psychotic disorders worldwide are ascribable to hospitalisation [6].

With its potential disadvantages, it is important to be aware of the factors that are associated with an increased risk of hospitalisation in FEP. A number of studies and reviews have looked at demographic variables predicting hospital admission, with Black African individuals significantly more likely to be admitted and remain in hospital for longer with a FEP [2, 3, 7–9]. There is a particular emphasis on ethnicity in the literature, but other demographic factors have also been studied. Substance misuse and longer duration of untreated psychosis were both associated with higher rates of hospitalisation [1], while older age of illness onset and the presence of a stable relationship were associated with lower rates [3].

While these studies have addressed some important factors, there remains a paucity of research on demographic and clinical determinants of hospitalisation in FEP. As there may be potential confounding between these factors, it would be required to examine all potential factors collectively in a sufficiently large cohort. By furthering our understanding of the predictors of admission, it would be possible to identify individuals who are at high risk for hospitalisation and then potentially develop interventions aimed at providing alternative methods to deliver care instead of admission. Therefore, this study aimed to (i) determine the proportion of individuals with a FEP who are admitted to hospital at the time of presentation (voluntary and involuntary admissions) (ii) identify demographic and clinical factors associated with admission to hospital.

## Methods

### Study design

This is a cohort study, consisting of 1220 young individuals who received treatment with the Early Psychosis Prevention and Intervention Centre (EPPIC) service, during the six year period from 1st January 2011 to 31st December 2016.

### Setting

This study was conducted at Orygen, which is the State Government funded mental health service for young people aged between 15 and 24, living within a defined catchment area covering approximately one million individuals in north western Melbourne, Australia. Orygen has its own dedicated acute psychiatric inpatient unit consisting of 16 beds, which provides care for individuals aged from 18 to 25 years. Young people aged 15 to 17 who required inpatient care were admitted to the psychiatric ward at the Royal Children’s Hospital in Melbourne. In addition, as there was a high demand for acute inpatient beds within the large catchment area, if the Orygen inpatient unit was full, young people over the age of 18 would be admitted to another adult psychiatric unit in the catchment area. Therefore, young people attending Orygen could have been admitted to a number of inpatient units across Melbourne. Even if admitted to other units, these young people would still be referred to Orygen and the relevant clinical team at Orygen would be informed about these admissions. Additionally, it would be recorded in the clinical notes that they were admitted to a different unit, which would then send on a discharge letter, which would be stored in the Orygen clinical file, meaning that all admissions from the other inpatient units could be readily recorded in this study.

The Early Psychosis Prevention and Intervention Centre (EPPIC) is one of the specialist streams within Orygen that provides comprehensive care to young people experiencing a first episode of psychosis. With a strong emphasis on treatment in the community, the service also provides intensive home based interventions. EPPIC provides treatment to approximately 200 new cases of FEP per year and receives direct referrals from the affected young person, their friends, family or caregivers, other mental health services, general practitioners, support services within the community, and law enforcement agencies.

### Participants

Included in the study were all individuals aged between 15 and 24 years old presenting to the EPPIC who were diagnosed with a FEP. To meet criteria for FEP, the person must have experienced full threshold psychotic symptoms for at least seven days. Individuals with comorbid substance misuse, comorbid personality disorders, intellectual disability, or low English language proficiency were all eligible for inclusion. Regarding substance misuse, participants were asked specifically about use of cannabis and methamphetamine, as these are the most commonly used illicit substances in Australia [10]. Regarding personality disorders, these were recorded at presentation, but it was found that they were often underrepresented, and not systematically assessed for the full duration of the study. Therefore, there was insufficient data to include comorbid personality disorders as a clinical variable. Following 3 months of treatment, more specific diagnoses were made for those who were still engaged with the service. These were then subdivided into affective vs non affective psychoses. Regarding individuals who disengaged from the service before three months, ‘Unspecified FEP’ was used as the diagnostic descriptor.

### Instruments

Severity of psychotic symptoms was measured using the short form SAPS (Scale for the Assessment of Positive Symptoms) [11]. This is an instrument that evaluates positive psychotic symptoms in four domains (hallucinations, delusions, bizarre behaviour, and formal thought disorder). The short form SAPS is an unstandardised version of the standard SAPS instrument. The individual items were scored from 0 to 5, based on information obtained from patient files. Inter rater reliability was assessed across five different participants, and on all positive psychotic symptoms items, inter rater agreement ranged from 80 to 100%. The Health of the Nation Outcome Scales (HoNOS) was used to assess self harming and aggressive behaviour at entry to the service and it was completed by the mental health clinician who worked closest with the young person [12].

### Data sources

Following presentation to the service, average length of treatment was 18 months, and maximum was 2 years, unless the individual was aged less than 18 after two years of care, at which point they could continue to attend EPPIC until their 18th birthday. During the initial phase of treatment, patients were reviewed once weekly. Intervals were then generally lengthened to fortnightly, and so forth, as indicated by the individual’s recovery process. All clinical notes pertaining to each episode of care were stored in a single patient chart, comprising documentation by the treating consultant psychiatrist, registrar, case manager, and other allied healthcare professionals involved. An instrument was developed to aid extraction of relevant clinical data including information about demographics and course of treatment. Patient files were analysed from point of entry to Orygen up until two years or discharge from the EPPIC. From the routine registration form filled out by each patient, data was obtained on demographics including age, gender, address, birthplace, employment status, and marital status. From medical records, data was obtained on clinical characteristics including severity of psychopathology, aggressive behaviour at presentation, self harm, diagnosis at three months of treatment, comorbid substance misuse, and family history of psychotic illness. If relevant data were unavailable from the clinical notes, then those cases were not included in the analysis for the variable/s in question.

### Statistical analysis

From the total number of young people presenting with a FEP, demographic and clinical characteristics of those who were admitted at the time of presentation were compared with those who received outpatient treatment. The same comparison was done for voluntary and involuntary admissions.

Binary logistic regression was used to determine the odds ratios and 95% C.I. for the predictor variables for the outcome of being admitted or treated as an outpatient, and for the outcome of being admitted voluntarily or involuntarily.

Initially, all variables were examined with univariate analysis using binary logistic regression (i.e., admission being the outcome variable, and each of the predictor variables such as age, sex etc., were examined individually). All predictor variables that were statistically significant (p < 0.05) on univariate analysis were then examined together with a multivariate logistic regression model.

### Ethical approval

This study received ethical approval from the Royal Melbourne Hospital Human Research and Ethics Committee (QA2016141).

## Results

### Description of participants

A total of 1220 young people presented with a first episode of psychosis during the six year study period, and there was data available for 99% (n = 1208) as to whether the individual was admitted at the time of presentation. Among this cohort, 58.6% (n = 708) were male and 41.4% (n = 500) were female. The median age was 20 years (I.Q.R.17–22). The majority were born in Australia (74.2%, n = 896) and had never been married (95.6%, n = 1130). Just over half (53.4%, n = 638) were enrolled in education, employment, or training. Regarding substance misuse, 52.3% (n = 632) reported using cannabis, and 27.6% (n = 334) used methamphetamine. Regarding diagnosis, 28.6% (n = 324) of individuals had an affective FEP. Regarding family history, 18.4% (n = 222) reported having a first degree relative with a psychotic disorder.

### Proportion of young people with FEP admitted to hospital and predictors of admission

It was found that just over half of the cohort (50.2%, n = 606) were admitted to hospital at the time of presentation with a FEP. A comparison of the demographic and clinical characteristics of the group who were admitted and the group who received outpatient treatment is provided in Table 1.

**Table 1:** Demographic and clinical characteristics of study participants

	Total cohort (*N* = 1208)		Inpatient admission		Outpatient		Statistics	
	%	N	%	N	%	N	OR (95% C.I.)	p
Demographic characteristics	S.D	Mean	S.D	Mean	S.D	Mean		
Mean age, years ± S.D	2.8	19.6	2.6	20.1	2.9	19	1.15 (1.10–1.20)	< 0.001
Sex	%	N	%	N	%	N		
Male	58.6	708	63.2	383	54.0	325	1.46 (1.16–1.84)	< 0.001
Female	41.4	500	36.8	223	46.0	277		
Marital status								
Never married/de-facto	95.6	1130	95.2	561	96.0	569	0.85 (0.48–1.48)	0.554
Married or previously married/de-facto	4.4	52	4.8	28	4.0	24		
Work status								
In education/employment/training	53.4	638	48.0	289	58.9	349	1.55 (1.23–1.95)	< 0.001
Not in education/employment/training (NEET)	46.6	557	52.0	313	41.1	244		
Family history of psychosis in first degree relative								
Positive family history of psychosis	18.4	222	16.8	102	19.9	120	0.81 (0.61–1.09)	0.164
No family history of psychosis	81.6	986	83.2	504	80.1	482		
First generation migrant								
Migrant	25.8	312	30.4	184	21.3	128	1.62 (1.24–2.10)	< 0.001
Born in Australia	74.2	896	69.6	422	78.7	474		
Illness and clinical features								
DSM-IV diagnosis								
Non-affective psychotic disorder	71.4	810	67.9	392	75.0	418	0.71 (0.54–0.91)	0.008
Affective psychotic disorder	28.6	324	32.1	185	25.0	139		
	Median	I.Q.R	Median	I.Q.R	Median	I.Q.R		
Duration of untreated psychosis, weeks	8.0	2–40	4.0	1–14	13.0	2–52	0.994 (0.992–0.996)	< 0.001
Cannabis use at presentation	%	N	%	N	%	N		
Cannabis use	52.3	632	58.4	354	46.2	278	1.64 (1.30–2.06)	< 0.001
No cannabis use	47.7	576	41.6	252	53.8	324		
Methamphetamine use at presentation								
Methamphetamine use	27.6	334	31.0	188	24.3	146	1.41 (1.09–1.81)	0.009
No methamphetamine use	72.4	874	69.0	418	75.7	456		
Aggressive behaviour at presentation								
Aggressive behaviour	51.8	439	55.9	255	47.1	184	1.43 (1.09–1.87)	0.010
No aggressive behaviour	48.2	408	44.1	201	52.9	207		
Self-harm								
Self-harm	20.7	177	19.1	87	22.6	90	1.24 (0.89–1.73)	0.204
No self-harm	79.3	677	80.9	369	77.4	308		
Psychopathology at presentation	S.D	Mean	S.D	Mean	S.D	Mean		
Severity of hallucinations	1.8	2.6	1.8	2.4	1.7	2.8	0.88 (0.82–0.93)	< 0.001
Severity of delusions	1.7	3.1	1.5	3.5	1.8	2.6	1.39 (1.29–1.49)	< 0.001
Severity of bizarre behaviour	1.8	1.6	1.9	2.2	1.5	1	1.47 (1.37–1.58)	< 0.001
Severity of thought disorder	1.6	1.3	1.7	1.8	1.3	0.7	1.56 (1.44–1.70)	< 0.001

On univariate analysis, the following factors were associated with an increased risk of admission: older age (OR = 1.15, 95% CI [1.10, 1.20]), male sex (OR = 1.46, 95% CI [1.16, 1.84]), not being in education, employment, or training (OR = 1.55, 95% CI [1.23, 1.95]), being a first generation migrant (OR = 1.62, 95% CI [1.24, 2.10]), having an affective illness (OR = 0.71, 95% CI [0.54, 0.91]), cannabis use (OR = 1.64, 95% CI [1.30, 2.06]), methamphetamine use (OR = 1.41, 95% CI [1.09, 1.81]), aggressive behaviour (OR = 1.43, 95% CI [1.09, 1.87]), more severe delusions (OR = 1.39, 95% CI [1.29, 1.49]), more severe bizarre behaviour (OR = 1.47, 95% CI [1.37, 1.58]), and more severe formal thought disorder (OR = 1.56, 95% CI [1.44, 1.70]). In contrast, the following factors were associated with a reduced risk of admission: more severe hallucinations (OR = 0.88, 95% CI [0.82, 0.93]) and longer duration of untreated psychosis (DUP) (OR = 0.99, 95% CI [0.99, 0.10]) which can be interpreted as with each increase of one week in the DUP, the risk of admission decreased by 1%. No significant impact on rates of all admissions was found for marital status, family history, or self harm.

On multivariate analysis, when potential confounding factors were controlled for, the following factors remained significant: severity of delusions (OR = 1.25, 95% CI [1.12, 1.40]), severity of bizarre behaviour (OR = 1.22, 95% CI [1.10, 1.36]), severity of formal thought disorder (OR = 1.17, 95% CI [1.04, 1.32]), being a first generation migrant (OR = 1.75, 95% CI [1.17, 2.62]), aggressive behaviour (OR = 1.42, 95% CI [1.02, 1.99]), and the DUP (OR = 0.996, 95% CI [0.994, 0.999]). The following factors became non significant: older age, male sex, not being in education, employment, or training, having an affective illness, cannabis use, methamphetamine use, and severity of hallucinations. Results of the multivariate analysis are provided in Table 2.

**Table 2:** Multivariate analysis of predictive factors for admission (all admissions and involuntary)

	All admissions			Involuntary admissions		
	OR (95% C.I.)	Std Error	p	OR (95% C.I.)	Std Error	p
Demographic characteristics						
Older age	1.06 (0.99–1.13)	0.03	0.096	1.10 (1.02–1.18)	0.04	0.008
Male sex	1.10 (0.76–1.58)	0.19	0.621	1.49 (1.01–2.20)	0.20	0.042
Work status	1.14 (0.80–1.63)	0.18	0.483	1.13 (0.78–1.63)	0.19	0.527
Family history of psychosis in first degree relative	0.91 (0.60–1.40)	0.22	0.678	0.92 (0.59–1.45)	0.23	0.732
Being a first generation migrant	1.75 (1.17–2.62)	0.21	0.007	1.63 (1.08–2.45)	0.21	0.019
Illness and clinical features						
DSM-IV diagnosis (affective vs non-affective)	0.75 (0.51–1.11)	0.20	0.150	0.77 (0.51–1.14)	0.20	0.188
Duration of untreated psychosis, weeks	0.996 (0.994–0.999)	0.001	0.006	0.10 (0.99–1.00)	0.002	0.025
Cannabis use at presentation	1.08 (0.73–1.59)	0.20	0.697	0.98 (0.65–1.47)	0.21	0.926
Methamphetamine use at presentation	1.28 (0.84–1.96)	0.22	0.245	1.37 (0.90–2.09)	0.22	0.148
Aggressive behaviour at presentation	1.42 (1.02–1.99)	0.17	0.040	1.42 (0.10–2.03)	0.18	0.053
Self-harm				0.87 (0.55–1.39)	0.24	0.571
Psychopathology at presentation						
Severity of hallucinations	0.96 (0.87–1.05)	0.05	0.348	0.92 (0.83–1.01)	0.05	0.090
Severity of delusions	1.25 (1.12–1.40)	0.06	0.000	1.32 (1.17–1.50)	0.06	0.000
Severity of bizarre behaviour	1.22 (1.10–1.36)	0.05	0.000	1.16 (1.05–1.29)	0.05	0.005
Severity of thought disorder	1.17 (1.04–1.32)	0.06	0.010	1.21 (1.08–1.36)	0.06	0.002

The predictor variables examined in this model accounted for approximately 27% of the variance in the admissions

### Proportion admitted involuntarily and predictors of involuntary admission

There was complete data in relation to the legal status of the admission for 91.7% (n = 556) of those admitted to hospital at the time of presentation. Of those admitted at presentation, 70.1% (n = 390) were admitted involuntarily and this represented 33.7% of the total cohort with a FEP.

On univariate analysis, the following factors were associated with an increased risk of involuntary admission: older age (OR = 1.17, 95% CI [1.12, 1.22]), male sex (OR = 1.88, 95% CI [1.46, 2.43]), not being in education, employment, or training (OR = 1.82, 95% CI [1.42, 2.33]), being a first generation migrant (OR = 1.64, 95% CI [1.25, 2.15]), having an affective illness (OR = 0.73, 95% CI [0.60, 0.97]), cannabis use (OR = 1.75, 95% CI [1.36, 2.24]), methamphetamine use (OR = 1.67, 95% CI [1.28, 2.18]), aggressive behaviour (OR = 1.52, 95% CI [1.15, 2.02]), self harm (OR = 1.66, 95% CI [1.15, 2.38]), more severe delusions (OR = 1.54, 95% CI [1.41, 1.68]), more severe bizarre behaviour (OR = 1.45, 95% CI [1.35, 1.56]), and more severe formal thought disorder (OR = 1.57, 95% CI [1.45, 1.70]). The following factors were associated with a reduced risk of involuntary admission: more severe hallucinations (OR = 0.84, 95% CI [0.78, 0.90]) and longer DUP (OR = 0.993, 95% CI [0.991, 0.996]). No association with involuntary admission rates was found for marital status or family history.

On multivariate analysis, when potential confounding factors were controlled for, the following factors remained significant: older age (OR = 1.10, 95% CI [1.02, 1.18]), male sex (OR = 1.49, 95% CI [1.01, 2.20]), severity of delusions (OR = 1.32, 95% CI [1.17, 1.50]), severity of bizarre behaviour (OR = 1.16, 95% CI [1.05, 1.29]), severity of formal thought disorder (OR = 1.21, 95% CI [1.08, 1.36]), being a first generation migrant (OR = 1.63, 95% CI [1.08, 2.45]) and the DUP (OR = 0.10, 95% CI [0.99, 1.00]). The following factors became non significant: not being in education, employment, or training, having an affective illness, cannabis use, methamphetamine use, aggressive behaviour, self harm, and severity of hallucinations. These results are provided in Table 3.

**Table 3:** Demographic and clinical characteristics of study participants associated with involuntary admission at presentation

	Involuntary admission		Voluntary treatment		Statistics	
	%	N	%	N	OR (95% C.I.)	p
Demographic characteristics	S.D	Mean	S.D	Mean		
Mean age, years ± S.D	2.5	20.4	2.9	19.2	1.17 (1.12–1.22)	< 0.001
Sex	%	N	%	N		
Male	67.9	265	53.3	409	1.88 (1.46–2.43)	< 0.001
Female	32.1	125	46.7	359		
Marital status						
Never married/de-facto	95.8	363	95.6	724	1.05 (0.57–1.94)	0.865
Married or previously married/de-facto	4.2	16	4.4	33		
Work status						
In education/employment/training	43.8	170	58.7	445	1.82 (1.42–2.33)	< 0.001
Not in education/employment/training (NEET)	56.2	218	41.3	313		
Family history of psychosis in first degree relative						
Positive family history of psychosis	16.9	66	19.0	146	0.87 (0.63–1.20)	0.391
No family history of psychosis	83.1	324	81.0	622		
First generation migrant						
Migrant	32.3	126	22.7	174	1.64 (1.25–2.15)	< 0.001
Born in Australia	67.7	264	77.3	594		
Illness and clinical features						
DSM-IV diagnosis						
Non-affective psychotic disorder	67.0	252	73.6	523	0.73 (0.56–0.97)	0.027
Affective psychotic disorder	33.0	124	26.4	188		
	Median	I.Q.R	Median	I.Q.R		
Duration of untreated psychosis, weeks	4.0	1–12	12.0	2–52	0.993 (0.991–0.996)	< 0.001
Cannabis use at presentation	%	N	%	N		
Cannabis use	61.3	239	47.1	362	1.75 (1.36–2.24)	< 0.001
No cannabis use	38.7	151	52.9	406		
Methamphetamine use at presentation						
Methamphetamine use	34.9	136	24.2	186	1.67 (1.28–2.18)	< 0.001
No methamphetamine use	65.1	254	75.8	582		
Aggressive behaviour at presentation						
Aggressive behaviour	58.0	184	47.7	244	1.52 (1.15–2.02)	0.004
No aggressive behaviour	42.0	133	52.3	268		
Self-harm						
Self-harm	15.9	50	23.8	124	1.66 (1.15–2.38)	0.007
No self-harm	84.1	265	76.2	397		
Psychopathology at presentation	S.D	Mean	S.D	Mean		
Severity of hallucinations	1.9	2.2	1.7	2.7	0.84 (0.78–0.90)	< 0.001
Severity of delusions	1.4	3.8	1.8	2.7	1.54 (1.41–1.68)	< 0.001
Severity of bizarre behaviour	1.9	2.4	1.6	1.2	1.45 (1.35–1.56)	< 0.001
Severity of thought disorder	1.7	2.0	1.4	0.8	1.57 (1.45–1.70)	< 0.001

## Discussion

### Summary of findings

There are a number of important findings from this study. First, there was a high admission rate for individuals presenting with FEP, with over half being admitted. Second, there were a number of factors that predicted all admissions, namely migrant status, aggressive behaviour, and severity of psychotic symptoms. Regarding involuntary admissions specifically, migrant status and symptom severity remained significant, but aggressive behaviour did not. Male sex and older age were also significant factors for involuntary admissions. Both for all admissions and for involuntary admissions, a longer DUP was associated with lower admission rates.

### Comparison to previous literature

Results from this study are consistent with previous literature in that there remains a relatively high admission rate among people presenting with FEP [1, 3]. However, the rate is lower than that demonstrated in a recent systematic review comprising 134,100 patients with established schizophrenia, where overall voluntary and involuntary admission rates were 61.9% and 43.0%, respectively [13].

It has been demonstrated that migrants and ethnic minorities are more likely to be admitted involuntarily for the treatment of a psychotic disorder [14], which this study replicated. The reason for this increased risk for involuntary admissions in migrants and ethnic minorities is not yet understood, but it has been hypothesized that it may be due to this population having greater barriers to accessing care through primary care and being more likely to have police involved in their pathway to care and a higher perceived risk of violence [14].

Another interesting finding from this study is that a longer DUP was associated with a decreased risk of being admitted to hospital and this is in contrast to previous findings [1]. A possible explanation for this finding is that insidious presentations (i.e. longer DUP, milder psychotic symptoms, less aggression) are less likely to result in admission. Therefore, considering these findings, it is likely that a two pronged approach is required. First, it is still required to continue endeavours to reduce the DUP in individuals with a FEP, as a longer DUP is a poor prognostic factor in terms of number of relapses and response to treatment, with less symptomatic improvement observed over time with a longer DUP [15]. Due to the increased risk of persistent illness in this group, consideration should be given for hospital treatment or assertive outreach, even if the presentation does not appear as severe. However, in addition to this, for individuals with a short DUP who have an acute presentation, alternatives to hospital need to be developed to avoid admission by default.

Of note, this study found that the association between substance misuse and admission became non significant when other factors were controlled for. Substance misuse has previously been associated with higher admission rates [1] in a study which also controlled for confounding factors. For those who do use illicit substances, they would be at increased risk for illness progression and subsequent relapse [16]. Therefore, they would be an important group to target with intensive outpatient follow up if not admitted. This may involve more of an assertive outreach approach or the implementation of addiction-focused strategies into their care plan.

Interesting to note is that self harm was not associated with admission on multivariate analysis. In a study by Harvey et al., the risk of self harm during the period of untreated first episode psychosis was associated with increased insight [17], which could be one possible explanation for this finding. Again this would be an important symptom to target in an outpatient care model given that self harm is a strong predictor of suicide in psychotic disorders [18], particularly in the early years subsequent to diagnosis.

In this study, male gender was associated with a higher rate of involuntary admission. Studies have shown that in general, male inpatients are significantly more likely to try and abscond than females from psychiatric units [19], suggesting they may be less inclined to comply with voluntary admission, although this was not specific to a FEP cohort.

### Clinical implications

There are two potential approaches to reducing hospital admission rates for people affected by a FEP. The first is to implement service level interventions that prevent the need for admission and the second is to have alternative methods of providing an equivalent level of care as to hospital admission.

A core component of early intervention for psychosis services is both primary and secondary prevention. Ultra high risk for psychosis clinics have been established with the aim of identifying young people at higher risk of developing a psychotic disorder and providing them with evidence based treatments for any concurrent disorders and to prevent the progression to a full threshold psychotic disorder [20]. There is also a secondary prevention component to the ultra high risk clinics, and it has recently been demonstrated that the risk for admission is nearly halved in young people with a FEP who initially presented via an ultra high risk clinic compared with presenting to the FEP service directly [21]. This finding was replicated in a subsequent study, which found that rates of both voluntary and involuntary hospital admissions were significantly reduced for young people with a FEP who previously attended another early intervention service [22]. Therefore, while there has been considerable debate as to the effectiveness of the ultra high risk clinics [23], preventing admissions is an often overlooked benefit of these services.

In relation to providing alternatives to hospital admission, assertive outreach treatment is effective in providing flexible treatment in the community for individuals with early psychosis [24] and was associated with a significant reduction in hospital admissions [25]. At Orygen, there have been several new service developments to address this need.

In 2020, a ‘Hospital in The Home’ model of care was set up with the aim of providing an alternative, equivalent level of care to inpatient admission for young people who would ordinarily require hospitalisation. Through this method, the patient receives intensive, evidence based treatment within their home, maintaining the familiarity of their everyday life and keeping the individual and their family central in care planning.

In 2022, two new services started, The Orygen Youth Prevention and Recovery Centre and Youth Hospital Outreach Post-suicidal Engagement team. The Youth Prevention and Recovery Centre is a short-term, recovery-focussed residential service providing a youth friendly therapeutic environment experiencing significant mental health problems who are either leaving an acute hospital inpatient setting or who would benefit from 24-h a day support to avoid a hospital admission or as an early intervention. This residential service for up to 28 days and aims to help the young person and their treating community team to achieve recovery goals and support their treatment.

The Orygen Youth Hospital Outreach Post-Suicidal Engagement team aims to provide intensive, person-centred support for young people presenting to the ED following suicide attempts or new onset suicidal ideation. Eligible young people are contact within 24 h of referral, and receive support for up to three months with a team of mental health clinicians and lived experience workforce. A holistic approach aims to help young people address factors that contribute to stress and distress in their lives across psychological, family, psychosocial domains.

For future research, it would be important to obtain qualitative data on the experiences of all those involved regarding the different treatment pathways. Admission is a clinical decision to be made in varying settings including primary care centres, emergency departments, and outpatient departments. It would be of interest to obtain structured feedback from patients, families, and staff about their impressions of inpatient vs outpatient care.

### Strengths and limitations

There are a number of strengths to this study, in that it is a large, representative cohort of treated cases of FEP, in which there were no exclusion criteria, and it was possible to examine and control for a range of demographic and clinical factors. However, the findings need to be considered with the limitations of the study. First, while the severity of psychopathology was scored using a structured instrument and inter rater reliability was performed, the researchers had to score the severity of symptoms from clinical notes, and the instrument used was the unstandardised short form SAPS. In addition, the relatively young median age of this cohort could limit the capacity to generalise findings to other FEP groups. Furthermore, while a large number of demographic and clinical factors were examined and controlled for in this study, there was no information pertaining to other factors that may have been associated with the decision for a young person to be admitted to hospital, such as the level of social supports within the family and also the individual’s preference as to where to receive treatment.

## Conclusions

This study highlights the high admission rate for FEP, even in a well established early intervention service. Three strong predictors of admission in FEP were identified, namely severity of positive symptoms, except hallucinations, a shorter DUP and migrant status. In order to reduce the admission rate in this population, interventions need to be provided at multiple levels. First, identifying individuals who may be ultra high risk for psychosis or experiencing a prodromal state, and intensifying their treatment plan within the community may reduce the risk of an acute deterioration and the requirement for hospitalisation. Second, for those who do reach the stage of presenting acutely, we need to have an alternative to hospital admission in place, that is structured and resourced adequately enough to meet their therapeutic and diagnostic needs.

When confounders were controlled for, severity of psychopathology remained one of the strongest predictors of being admitted, and of being admitted involuntarily, at the time of presentation. These findings emphasise the need for action before people reach the severe stage of psychosis. By focusing on early intervention and identifying Ultra high risk individuals, we can tailor service delivery in FEP, with the aim of preventing deterioration to the severe stage. In doing so, we can reduce acute admissions and continue to move towards community based treatment for mental health disorders, where clinically appropriate.
